# A Simple Precursor for Highly Functionalized Fused Imidazo[4,5-*b*]pyridines and Imidazo[4,5-*b*]-1,8-naphthyridine

**DOI:** 10.3390/molecules21121646

**Published:** 2016-12-01

**Authors:** Omar K. Al-duaij, Magdi E.A. Zaki, Abdel-Rhman B. A. El Gazzar

**Affiliations:** 1 Chemistry Department, College of Science, IMSIU (Al-Imam Mohammad Ibn Saud Islamic University), Riyadh 11623, Saudi Arabia; abagazzar@imamu.edu.sa; 2 Photochemistry Department, National Research Centre, Cairo 12622, Egypt

**Keywords:** imidazo[4,5-*b*]pyridine, diaminomaleonitrile, carbon acid, imidazo[4,5-*b*]-1,8-naphthyridine, malomonitrile, 2-amino-1,1,3-propenetricarbonitrile

## Abstract

1-alkyl aryl-5-amino-4-(cyanoformimidoyl)imidazoles **4** were reacted with malononitrile and 2-amino-1,1,3-propenetricarbonitrile under mild experimental conditions, which led to 5-amino-3-(substituted benzyl)-6,7-dicyano-3*H*-imidazo[4,5-*b*]pyridines **5** and 6,8-diamino-3-(4-substituted benzyl)-3*H*-imidazo[4,5-*b*]-1,8-naphthyridine-7,9-dicarbonitrile **6**, respectively, when the reaction was carried out in the absence of a base, or to 5,7-diamino-3-(4-alkyl aryl)-3*H*-imidazo[4,5-*b*]pyridine-6-carbonitrile **8**, and 6,8,9-triamino-3-(4-substitutedbenzyl)-3*H*-imidazo[4,5-*b*]-1,8-naphthyridine-7-carbonitrile **10** in the presence of 1,8-diazabicyclo(5.4.0)undec-7-ene (DBU). Both reactions evolved from an adduct formed by nucleophilic attack of the malononitrile anion or 2-amino-1,1,3-propenetricarbonitrile anion to the carbon of the cyanoformimidoyl substituent. In the case of the malononitrile anion, a 5-amino-1-alkyl aryl-4-(1-amino-2,2-dicyanovinyl)imidazole **7** was isolated when this reaction was carried out in the presence of DBU. The structure of compound **7** was confirmed by spectroscopic methods, and cyclized intramolecularly to **8** by heating in ethanol/triethyl amine.

## 1. Introduction

The imidazo[4,5-*b*]pyridine ring system **5**, **8** represent a core skeleton of a class of heterocyclic compound analogues of purine with their diversity of biological activity [1,2,3,4,5], depending on the substituents on the pyridine ring. Imidazo[4,5-*b*]pyridine has been proved to be a useful scaffold for medicinal agents [6,7]. It has been evaluated as an antagonist of various biological receptors, including angiotensin-II [8,9], platelet-activating factor (PAF) [10,11], and metabotropic glutamate subtype V [12]. Substituted imidazo[4,5-*b*]pyridines have also been explored for their potential as anticancer [13,14], inotropic, [15,16], and selective antihistamine (H1) agents [17]. Imidazo[4,5-*b*]pyridine derivatives are also reported as Aurora kinases [18] and cyclic phosphodiesterase inhibitors [19]. Recently, the synthesis of imidazo[4,5-*b*]pyridine derivatives has been one of the interests in the field of organic synthesis. Synthesis of imidazo[4,5-*b*]pyridine mainly depends on the generation of the imidazole moiety from a substituted pyridine under appropriate reaction conditions. Building a pyridine ring from readily available substitute imidazole is rare [20].

In our research group, we are interested in exploring the reactivity of 5-amino-4-(cyanoformimidoyl)imidazoles **4**, easily formed by base-catalyzed cyclization of 2-amino-1,2-dicyano formamidines **3** [21]. Cyanoformimdoyl imidazole **4** is considered a straightforward and convenient precursor for fused nitrogen heterocycles [22,23,24,25,26]. The generation of fused imidazoles is initiated at a cyanoformimidoyl unit in the 4-position of the imidazole ring [20]. This work reports the first simple synthesis of *N*-alkyl aryl imidazo[4,5-*b*]pyridines, and *N*-alkyl aryl imidazo[4,5-*b*]1,8-naphthyridine isolated from the reaction of cyanoformimdoyl imidazole **4** with carbon acid derivatives, namely malononitrile and 2-amino-1,1,3-propenetricarbonitrile, under appropriate experimental conditions.

## 2. Results and Discussion

**Scheme 1:**
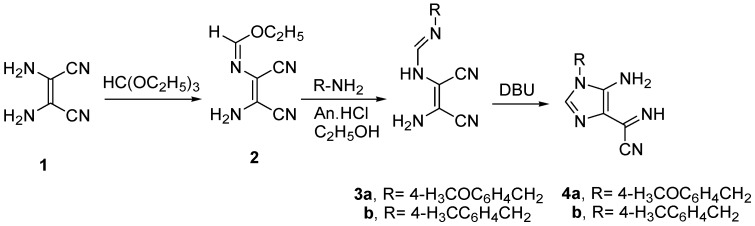
Synthesis of 5-amino-1-(substituted benzyl)-4-cyanoformimidoyl imidazole **4**.

Heating diaminomaleonitrile **1** with triethyl orthoformate in dioxane afforded ethyl [2-amino-1,2-dicyanovinyl] imidoformate **2** [21]. Treatment of **2** with appropriate amines, namely 4-methoxybenzylamine and 4-methylbenzylamine catalyzed by aniline hydrochloride, formed (substituted benzyl)-*N*-(2-amino-1,2-dicyanovinyl)formimidine **3** which underwent intramolecular cyclization in the presence of 1,8-diazabicyclo(5.4.0)undec-7-ene (DBU) to form 5-amino-1-(substituted benzyl)-4-cyanoformimidoyl imidazole derivative **4** [27] (Scheme 1 and Section 3).

**Scheme 2:**
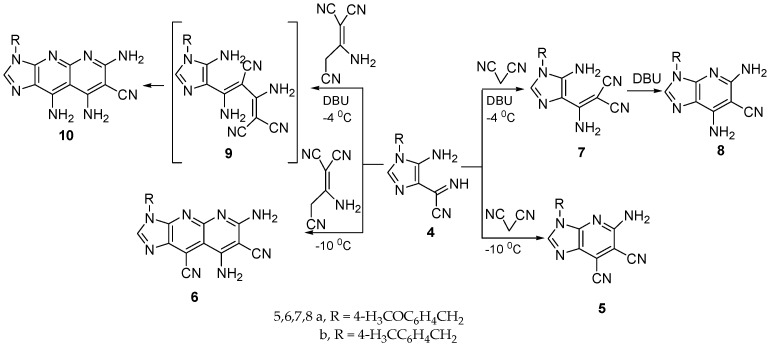
Synthesis of Fused imidazole **5**, **6**, **8**, **10**.

The combination of cyanoformimdoyl imidazole **4** with malononitrile or 2-amino-1,1,3-propenetricarbonitrile takes place to give imidazo[4,5-*b*]pyridine **5** and imidazo[4,5-*b*]-1,8-naphthyridine **6** in good yield, upon treatment of the suspended mixture in acetonitrile/ethanol (3:1) for 8 h at 0 °C, followed by five days at −10 °C (Scheme 2). The optimal reaction conditions were necessary to keep the concentration of the evolved ammonia in the reaction medium until total consumption of the starting materials.

Spectroscopic methods confirmed the structure of imidazo[4,5-*b*]pyridine **5a**; its IR spectrum shows the two weak bands of the cyano groups at 2237 cm^−1^ and a medium intensity peak at 2119 cm^−1^. The presence of the two cyano groups is confirmed by its ^13^C-NMR, as two peaks are present around δ 114.1 and 115.2 ppm. The chemical shifts for C-5, C-6, and C-7 are around δ 157.8, 86.4, and 112.9 ppm respectively. The presence of the amino group is confirmed in the ^1^H-NMR spectrum as a singlet at δ 7.36 ppm, integrating for two protons, and at 8.82 ppm, integrating for one proton corresponding to CH-imidazole. Moreover, the structure imidazo[4,5-*b*] [1,8] naphthyridine **6a** was confirmed by spectroscopic methods; its IR spectrum shows the two bands of the cyano groups at 2226 cm^−1^ and a sharp intensity peak at 2212 cm^−1^, which is located between two amino groups. The presence of the two cyano groups is confirmed by its ^13^C-NMR, as two peaks are present around δ 115.2 and 117.3 ppm. The chemical shifts for C-6, C-7, and C-8 around are δ 158.7, 85.9, and 109.8 ppm, respectively. The presence of the two amino groups is confirmed by the ^1^H-NMR spectrum as a broad singlet at δ 6.48 and 7.29 ppm, integrating for two protons each.

**Scheme 3:**
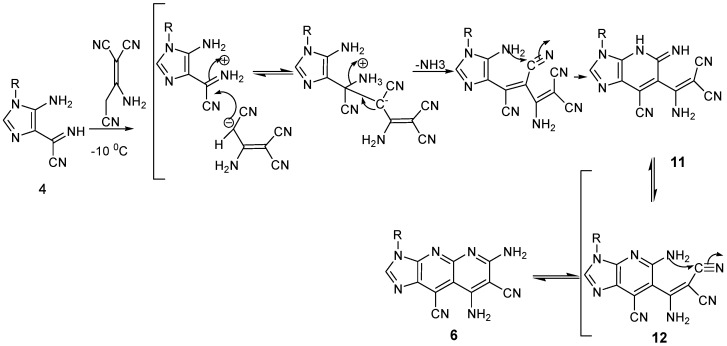
Proposed mechanism for Synthesis Fused imidazole **6** in absence of DBU.

Recently, Zaki et al. reported a mechanism for the reaction of *N*-aryl cyanoformimdoyl imidazole **4** with malononitrile, in the absence of a base [20]. The imino nitrogen of imidazole **4** may act as a mild base, and a positive charge generated in the imino nitrogen accelerates the nucleophile attack by the 1,1,3-tricyanopropene anion, leading to the elimination of ammonia and the formation of intermediate **11**, which rapidly cyclizes in situ by successive intramolecular cyclization, affording imidazo[4,5-*b*]-1,8-naphthyridine **6** in good yield (Scheme 3). Cyclization of intermediate **11** to **12** may be catalyzed with the evolved ammonia to give **6**. 

**Scheme 4:**
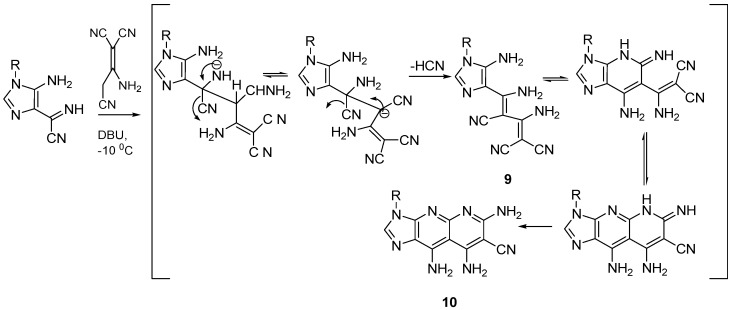
The Proposed mechanism for Synthesis Fused imidazole **10** in presence of DBU.

In the presence of DBU as a strong base, in situ generated anions of malononitrile or 1,1,3-propentricarbonitrile reacted with imidazole **4** in a mixture of acetonitrile/ethanol (1:3) under conditions similar to those of the reaction in the absence of DBU to give **7** and **10** in good yield. We found the amount of DBU used must be in excess to confirm the total generation of a carbanion. Intramolecular cyclization of dicyano imidazole derivative **7** afforded imidazo[4,5-*b*]pyridine **8** in moderate yield via refluxing in the presence of triethylamine as a catalyst (Scheme 4 and Section 3).

The structure of imidazole **7b** was confirmed by spectroscopic methods; its IR spectrum shows the two cyano groups at 2210 cm^−1^ and a medium intensity peak at 2191 cm^−1^. The presence of the two cyano groups is confirmed by its ^13^C-NMR, as two weak peaks are present around δ 125.2 and 117.8 ppm. Both carbon atoms of the alkene substituents in the 4-position of the imidazole ring are characterized by their position in the ^13^CNMR. The carbon atom directly bonded to the amino group gives a signal of δ 162.9 ppm, while the adjacent carbon bonded to the two cyano groups appeared as a weak signal of δ 44.5 ppm. In the ^1^H-NMR spectrum, the two amino groups appear as two singlets at δ 5.88 ppm, and 7.82 ppm, integrated for two protons for each one. Moreover, the structure of imidazo[4,5-*b*]pyridine **8a** was confirmed by spectroscopic methods; its IR spectrum shows a sharp intensity of a cyano group at 2212 cm^−1^, which is located between two amino groups. The presence of the cyano group is assigned by its ^13^C-NMR, as a signal of δ 115.4 ppm. The chemical shifts for C-5, C-6, and C-7 are around δ 159.6, 70.3, and 146.9 ppm, respectively. The presence of the two amino groups is visible both in the IR and in the ^1^H-NMR spectra, which shows two signals of δ 6.44 and 7.21 ppm, each one integrating for two protons. The formation of the fused imidazole ring is supported by the presence of one singlet of δ 8.21 ppm, a characteristic for C-H of the fused imidazole moiety [20]. The spectroscopic methods confirmed the structure of imidazo[4,5-*b*]-1,8-naphthyridine **10a**; its IR spectrum shows the strong band of a cyano group at 2211 cm^−1^. The presence of the cyano group is confirmed by its ^13^C-NMR, as a peak is present at δ110.6. The chemical shifts for C-6, C-7, and C-8 are around δ 156.9, 71.4, and 159.9 ppm, respectively. The presence of the amino groups is confirmed in the ^1^H-NMR spectrum as a three singlet at δ 7.32 ppm, 6.81 ppm, 6.69 ppm, respectively, integrating for two protons each, and at 8.96 ppm, integrating for one proton, corresponding to CH-imidazole.

The high concentration of malononitrile anions formed and the reaction with cyanoformimdoyl imidazole **4** lead to stable carbanions, which evolve into alkene derivatives **9** by the elimination of cyanide ions, yielding 4-dicyanovinyl imidazole derivative **7**. Moreover, we do believe that the formation of 2,4-diamino-4-substituted-imidazolyl-4-buta-1,3-diene-1,1,3-tricarbonitrile derivative **9** has been formed as an intermediate, followed by intramolecular cyclization in situ, affording imidaz[4,5-*b*]-1,8-naphridine **10** in moderate yield. The intramolecular cyclization was induced rapidly as a result of the presence of highly deficient alkene without a catalyst.

The acidity of malononitrile [28] could be a factor in isolating imidazole **7**, which cyclized intramolecularly in the presence of a catalytic amount of base to imidazo[4,5-*b*]pyridine **8**. Moreover, the acidity of 1,1,3-tricyanopropene induced the formation of imidazole derivative **9**, as a result of being highly substituted electron-deficient, forced the intramolecular cyclization, in situ without a base.

## 3. Experimental Section

### 3.1. General Information

All compounds were fully characterized by elemental analysis and spectroscopic data. The NMR spectra were recorded at 300 MHz for ^1^H and 75 MHz for ^13^C. Deuterated dimethyl sulfoxide (DMSO) was used as solvent. The chemical shifts are expressed in d (parts per million) and the coupling constants (*J*) in hertz (Hz). IR spectra were recorded on a FT-IR 400D Spectrophotometer (Shimadzu, Kyoto, Japan) using Nujol mulls and NaCl cells. The reactions were monitored by thin layer chromatography (TLC) using silica gel. The melting points were determined on a melting point apparatus (Stuart-SMP20, Staffordshire, United Kingdom) and are uncorrected.

#### Safety

The reaction mixtures containing cyanide ions can be oxidized with calcium hypochlorite in a basic solution, to much less toxic cyanate ions, following the destruction procedure described in the literature [29].

### 3.2. Synthesis of Ethyl N-(2-Amino-1,2-dicyanovinyl)formimidate ***2***

A mixture of diaminomaleonitrile (55.5 mmol, 1 equiv.) and triethyl orthoformate (55.5 mmol, 1 equiv.) in dioxane (80 mL) was heated at reflux in a flask fitted with a short Vigreux column, a distillation head, a condenser, and a receiver. Ethanol mixed with 1,4-dioxane was collected continuously until the temperature in the distillation head reached 99–100 °C (approximately 20 min). The clear brown liquid in the distillation pot was allowed to cool overnight. The reaction mixture was diluted with hot diethyl ether, filtered to remove the dark brown solid impurity, and left to cool overnight to give **2** as colorless needles (6.6 g, 84%). IR (Nujol): 3313, 2251, 2214, 1641 cm^−1^; ^1^H-NMR (300 MHz, DMSO-*d*_6_): δ = 1.36 (t, *J* = 7.3 Hz, 3H, CH_3_), 4.27 (q, *J* = 7.3 Hz, 2H, CH_2_), 4.66 (brs, 2H, NH_2_), 7.97 (s, 1H, CH). 

### 3.3. General Procedure for Synthesis of N-[2-Amino-1,2-dicyanovinyl]-N′-(4-substituted benzyl)imidoformamide ***3a**,**b***

The 4-substituted benzyl amine (6.70 mmol, 1.1 equiv) was added to a suspension of 2 (6.09 mmol, 1 equiv.) in dry EtOH which contained aniline hydrochloride (0.02 g). The mixture was stirred at room temperature until TLC showed that all the formimidate had disappeared (~3 h) and the pale yellow solid was obtained by filtration, washed with diethyl ether.

*N-[2-Amino-1,2-dicyanovinyl]-N′-(4-methoxybenzyl)imidoformamide* (**3a**) [27]. Off-white solid, (1.4g, 69%). m.p. 98–100 °C, IR (Nujol): 3311, 2229, 2211, 1637, 1591cm^−1^. ^1^H-NMR (300 MHz, DMSO-*d*_6_): δ = 3.74 (s, 3H, OCH_3_), 4.47 (d, *J* = 4.5 Hz, 2H, CH_2_), 6.11 (s, 2H, NH_2_), 6.93 (d, *J* = 8.2 Hz, 2H, Ar-H), 7.29 (d, *J* = 8.2 Hz, 2H, Ar-H), 7.75 (d, *J* = 2.3 Hz, 1H, CH), 8.17 (brs, 1H, NH). ^13^C-NMR (75 MHz, DMSO-*d*_6_): δ = 43.6, 55.7, 106.6, 114.1, 115.6, 116.8, 117.7, 129.9, 131.3, 151.0, 158.9. 

*N-[2-Amino-1,2-dicyanovinyl]-N′-(4-methylbenzyl)imidoformamide* (**3b**). Off-white solid, (1.2 g, 5.02 mmol, 65%). m.p. 109–110 °C, IR (Nujol): 3313 (N-H str.), 2222, 2211, 163, cm^−1^. ^1^H-NMR (300 MHz, DMSO-*d*_6_): δ = 2.16 (s, 3H, CH_3_), 4.33 (d, *J* = 4.2 Hz, 2H, CH_2_), 6.24 (s, 2H, D_2_O-exchangable NH_2_), 7.13 (d, *J* = 8.4 Hz, 2H, Ar-H), 7.28 (d, *J* = 8.4 Hz, 2H, Ar-H), 7.92 (d, *J* = 2.1 Hz, 1H, CH), 8.23 (brs, 1H, NH). ^13^C-NMR (75 MHz, DMSO-*d*_6_): δ = 18.8, 54.6, 104.4, 112.5, 114.6, 118.7, 127.6, 129.1, 134.7, 138.8, 153.4. Anal. Calcd for: C_13_H_13_N_5_: C, 65.25; H, 5.48; N, 29.27. Found C, 65.14; H, 5.37; N, 29.14.

### 3.4. General Procedure for Synthesis 5-Amino-1-(4-substituted benzyl)-4-cyanoformimidoyl imidazole ***4a**,**b***

To a suspension of **3** (4.2 mmol) in dry ethanol (10 mL), DBU was stirred 2 h at room temperature until the starting material was consumed. The precipitate that formed was filtered off, washed with diethyl ether, and dried to afford an off-white solid **4**.

*5-Amino-1-(4-methoxy benzyl)-4-cyanoformimidoyl imidazole* (**4a**). To a suspension of **3a** in dry EtOH (10 mL), DBU was added (one drop). The reaction mixture was stirred for 2 h at room temperature until the starting material was consumed (TLC). The precipitated product was filtered, washed with diethyl ether and dried to afford 4a as an off-white solid (1.35 g, 67%). m.p. 92–94 °C, IR (Nujol): 3297, 3131, 2227, 1636, 1549 cm^−1^. ^1^H-NMR (300 MHz, DMSO-*d*_6_): δ = 3.72 (s, 3H, OCH_3_), 5.03 (s, 2H, CH_2_), 6.76 (brs, 2H, D_2_O-exchangable NH_2_), 6.91 (d, *J* = 8.2 Hz, 2H, Ar-H), 7.22 (d, *J* = 8.2 Hz, 2H, Ar-H), 7.30 (s, 1H, CH-imidazole), 10.87 (s, 1H, D_2_O-exchangable NH). ^13^C-NMR (75 MHz, DMSO-*d*_6_): δ = 45.7, 55.7, 114.1, 114.6, 116.7, 128.8, 129.5, 132.8, 143.5, 144.7, 159.4. Anal. Calcd for: C_13_H_13_N_5_O: C, 65.25; H, 5.48; N, 29.27. Found C, 65.39; H, 5.48; N, 29.31.

*5-Amino-1-(4-methyl benzyl)-4-cyanoformimidoyl imidazole* (**4b**). Off-white solid (0.87 g, 3.6 mmol, 87%). m.p. 174–175 °C, IR (Nujol): 3313, 2222, 2211, 1639 cm^−1^. ^1^H-NMR (300 MHz, DMSO-*d*_6_): δ = 2.23 (s, 3H, CH_3_), 5.11 (d, 2H, CH_2_), 6.82 (s, 2H, D_2_O-exchangable NH_2_), 7.19 (d, *J* = 8.4 Hz, 2H, Ar-H), 7.33 (d, *J* = 8.4 Hz, 2H, Ar-H), 7.42 (s, 1H, CH-imidazole), 10.92 (brs, 1H, D_2_O-exchangable NH). ^13^C-NMR (75 MHz, DMSO-*d*_6_): δ = 22.4, 53.4, 116.2, 119.8, 128.8, 129.4, 135.3, 136.8, 138.2, 147.8, 151.4. Anal. Calcd for: C_13_H_13_N_5_: C, 65.25; H, 5.48; N, 29.27. Found C, 65.21; H, 5.27; N, 29.32.

### 3.5. General Procedure for the Synthesis of 5-Amino-3-(substituted benzyl)-6,7-dicyano-3H-imidazo[4,5-b]pyridines ***5a**,**b***

Malononitrile (0.4 g, 6.1 mmol) was added to a suspension of 5-amino-1-(substituted benzyl)-4-(cyanoformmidoyl) imidazole **4** (4.4 mmol) in a mixture of acetonitrile/ethanol (3:1). The addition took place in an ice bath, and the mixture was stirred in ice for 8 h. The reaction was allowed to stand at −10 °C for five days, when the solid was filtered and washed with diethyl ether and cold ethanol.

*5-Amino-3-(4-methoxybenzyl)-3H-imidazo[4,5-b]pyridine-6,7-dicarbonitrile* (**5a**). Off-white solid (1.1 g, 3..62 mmol, 82%). m.p. 336–337 °C, IR (Nujol): 2237, 2119, 1649, 1604, 1572, 1544 cm^−1^. ^1^H-NMR (300 MHz, DMSO-*d*_6_): δ = 3.92 (s, 3H, OCH_3_), 5.28 (s, 2H, CH_2_), 7.21 (d, *J* = 5.8 Hz, 2H, Ar-H),7.36 (s, 2H), 7.67 (d, *J* = 5.8 Hz, 2H, Ar-H), 8.82 (s, 1H) ^13^C-NMR (75 MHz, DMSO-*d*_6_): δ = 50.2, 55.8, 86.4, 112.9, 114.1, 115.2, 115.7, 125.7, 127.2, 128.4, 146.5, 149.5, 157.8, 159.4. Calcd for: C_16_H_12_N_6_ O: C, 63.15; H, 3.97; N, 27.62. Found C, 62.98; H, 3.79; N, 27.41.

*5-Amino-3-(4-methylbenzyl)-3H-imidazo[4,5-b]pyridine-6,7-dicarbonitrile* (**5b**). Off-white solid, (0.96 g, 3.33 mmol, 76%). m.p. 319–320 °C, IR (Nujol): 2240, 2122, 1643, 1605, 1584, 1549 cm^−1^. ^1^H-NMR (300 MHz, DMSO-*d*_6_): δ = 2.38 (s, 3H, CH_3_), 5.22 (s, 2H, CH_2_), 7.31 (s, 1H), 7.37 (d, *J* = 5.8 Hz, 2H, Ar-H), 7.59 (d, *J* = 5.8 Hz, 2H, Ar-H), 8.88 (s, 2H). ^13^C-NMR (75 MHz, DMSO-*d*_6_): δ = 21.2, 49.7, 86.4, 112.9, 114.1, 115.4, 124.3, 127.9, 129.8, 131.3, 138.4, 146.8, 149.8, 158.4. Calcd for: C_16_H_12_N_6_: C, 66.66; H, 4.20; N, 29.15. Found C, 66.72; H, 4.29; N, 29.28.

### 3.6. General Procedure for the Synthesis 6,8-Diamino-3-(4-substituted benzyl)-3h-imidazo [4,5-b]-1,8-naphthyridine-7,9-dicarbonitrile ***6***

The 2-amino-1,1,3-propenetricarbonitrile (0.08 g, 6 mmol) was added to a suspension of 5-amino-1-(substituted benzyl)-4-(cyanoformmidoyl)imidazole 4 (4.4 mmol) in a mixture of acetonitrile/ethanol (3:1). The addition took place in an ice bath, and the mixture was stirred in ice for 6 h. The reaction was allowed to stand at −10 °C for eight days, when the solid was filtered and washed with diethyl ether and cold ethanol.

*6,8-Diamino-3-(4-methoxylbenzyl)-3H-imidazo[4,5-b]-1,8-naphthyridine-7,9-dicarbonitrile* (**6a**). (0.98 g, 2.65 mmol, 60%). m.p. 351–352 °C, IR (Nujol): 2226, 2212, 1686, 1594 cm^−1^. ^1^H-NMR (300 MHz, DMSO-*d*_6_): δ = 3.96 (s, 3H, OCH_3_), 5.31 (s, 2H, CH_2_), 6.48 (s, 2H), 7.29 (s, 2H), 7.31 (d, *J* = 8.6 Hz, 2H, Ar-H), 7.73 (d, *J* = 8.6 Hz, 2H, Ar-H), 8.98 (s, 1H). ^13^C-NMR (75 MHz, DMSO-*d*_6_): δ = 49.9, 55.8, 85.9, 109.8, 118.4, 117.3, 118.1, 123.7, 128.4, 131.4, 138.7, 142.5, 149.9, 151.7, 158.7, 159.5, 160.2. Calcd for: C_19_H_14_N_8_: C, 61.62; H, 3.81; N, 30.25. Found C, 61.57; H, 3.85; N, 30.31.

*6,8-Diamino-3-(4-methylbenzyl)-3H-imidazo[4,5-b]-1,8-naphthyridine-7,9-dicarbonitrile* (**6b**). (0.94 g, 2.54 mmol, 58%). m.p. 340–342 °C, IR (Nujol): 2219, 2207, 1680, 1590 cm^−1^. ^1^H-NMR (300 MHz, DMSO-*d*_6_): δ = 2.36 (s, 3H, CH_3_), 5.41 (s, 2H, CH_2_), 6.48 (s, 2H), 7.29 (s, 2H), 7.47 (d, *J* = 8.6 Hz, 2H, Ar-H), 7.79 (d, *J* = 8.6 Hz, 2H, Ar-H), 8.96 (s, 1H). ^13^C-NMR (75 MHz, DMSO-*d*_6_): δ = 21.2, 50.1, 86.2, 110.6, 118.8, 121.4, 124.8, 128.7, 131.9, 137.4, 139.6, 140.5, 141.8, 149.2, 151.7, 156.1, 159.9. Calcd for: C_19_H_14_N_8_: C, 64.40; H, 3.98; N, 31.62. Found C, 64.29; H, 3.85; N, 31.57.

### 3.7. General Procedure for the Synthesis of 5-Amino-4-(1′-amino-2′,2′-dicyanovinyl)-1-(4-substituted benzyl)imidazole ***7***

DBU (0.53 mmol) was added to a solution of malononitrile (6.1 mmol) in acetonitrile/ethanol mixture (1:3) and kept in an ice bath. The mixture was sitirred in ice bath for 1 h. A suspension of cyanoformimdoyl imidazole **4a**,**b** (4.4 mmol) with the least amount of ethanol (2 mL) was added drop wise, and the mixture was stirred in the ice bath for 8 h. The reaction mixture was allowed to stand at −10 °C for three days, when the off-white solid was filtered and washed with ethanol and diethyl ether.

*5-Amino-4-(1′-amino-2′,2′-dicyanovinyl)-1-(4-methoxybenzyl)imidazole* (**7a**). (1.1 g, 3.7 mmol, 85%). m.p. 224–225 °C, IR (Nujol): 2221, 2198, 1658, 1625, 1581, 1544 cm^−1^. ^1^H NMR (300 MHz, DMSO-*d*_6_): δ = 3.82 (s, 3H, OCH_3_), 5.27 (s, 2H, CH_2_), 5.78 (s, 2H, NH_2_, D_2_O-exchangable NH_2_), 7.18 (d, *J* = 9 Hz, 2H, Ar-H), 7.38 (d, *J* = 9 Hz, 2H, Ar-H), 7.67 (d, 1H, CH-imidazole), 7.98 (brs, 2H, D_2_O-exchangable NH_2_). ^13^C-NMR (75 MHz, DMSO-*d*_6_): δ = 43.8, 55.4, 58.2, 111.3, 114.7, 115.1, 118.7, 126.9, 127.7, 134.1, 141.8, 159.8, 163.7. Anal. Calcd for: C_15_H_14_N_6_O: C, 61.21; H, 4.79; N, 28.55. Found C, 61.05; H, 4.61; N, 28.39.

*5-Amino-4-(1′-amino-2′,2′-dicyanovinyl)-1-(4-methylbenzyl)imidazole* (**7b**). (0.98 g, 3.5 mmol, 80%). m.p. 234–235 °C, IR (Nujol): 2210 , 2191; 1684; 1635; 1580; 1529 cm^−1^. ^1^H-NMR (300 MHz, DMSO-*d*_6_): δ = 2.32 (s, 3H, CH_3_), 5.22 (s, 2H, CH_2_), 5.88 (s, 2H, NH_2_, D_2_O-exchangable NH_2_), 7.37 (d, *J* = 8.7 Hz, 2H, Ar-H), 7.45 (d, *J* = 8.7 Hz, 2H, Ar-H), 7.62 (d, 1H, CH-imidazole), 7.82 (brs, 2H, D_2_O-exchangable NH_2_). ^13^C-NMR (75 MHz, DMSO-*d*_6_): δ = 21.3, 44.5, 50.4, 117.8, 0o125.2, 130.1, 132.0, 133.2, 138.3, 142.1, 159.1, 162.9. Anal. Calcd for: C_15_H_14_N_6_: C, 65.74; H, 5.52; N, 28.75. Found C, 65.65; H, 5.43; N, 28.88.

### 3.8. General Procedure for the Synthesis of 5,7-Diamino-3-substituted benzyl-6-cyano-3H-imidazo[4,5-b]pyridines ***8***

A suspension of 5-amino-4-(1′-amino-2′,2′-dicyanovinyl)-1-(4-substituted benzyl)imidazole **5** (3 mmol) in ethanol (5 mL) was combined with a catalytic amount of triethyl amine and the mixture was refluxed for 1 h. An off white precipitate formed, which was filtered and washed with ethanol and diethyl ether. 

*5,7-Diamino-3-(4-methoxybenzyl)-3H-imidazo[4,5-b]pyridine-6-carbonitrile* (**8a**). (0.72 g, 2.45 mmol, 82%). m.p. 321–322 °C, IR (Nujol): 2212, 1685, 1596, 1590 cm^−1^. ^1^H-NMR (300 MHz, DMSO-*d*_6_): δ = 3.91 (s, 3H, OCH_3_), 5.32 (s, 2H, CH_2_), 6.44 (s, 2H) 7.21 (s, 2H), 7.09 (d, *J* = 9 Hz, 2H, Ar-H), 7.76 (d, *J* = 9 Hz, 2H, Ar-H), 8.21 (s, 1H, CH-imidazole). ^13^C-NMR (75 MHz, DMSO-*d*_6_): δ = 49.5, 55.6, 70.3, 114.7, 115.4, 117.6, 126.1, 128.6, 137.7, 146.9, 150.3, 157.9, 159.6. Anal. Calcd for: C_15_H_14_N_6_ O: C, 61.21; H, 4.79; N, 28.55. Found C, 60.98; H, 4.63; N, 28.41.

*5,7-Diamino-3-(4-methylbenzyl)-3H-imidazo[4,5-b]pyridine-6-carbonitrile* (**8b**). (0.74 g, 2.66 mmol, 89%). m.p. 308–309 °C, IR (Nujol): 2204, 1674, 1585, 1580 cm^−1^. ^1^H-NMR (300 MHz, DMSO-*d*_6_): δ = 2.38 (s, 3H, CH_3_), 5.29 (s, 2H, CH_2_), 6.39 (s, 2H), 7.17 (s, 2H), 7.41 (d, *J* = 8.4 Hz, 2H, Ar-H), 7.71 (d, *J* = 8.4 Hz, 2H, Ar-H), 8.41 (s, 1H, CH-imidazole). ^13^C-NMR (75 MHz, DMSO-*d*_6_): δ = 21.3, 49.5, 70.1, 116.2, 118.1, 123.4, 128.9, 132.0, 136.8, 146.3, 152.6, 160.1. Anal. Calcd for: C_15_H_14_N_6_: C, 64.73; H, 5.07; N, 30.29. Found C, 64.59; H, 5.11; N, 30.37.

### 3.9. General Procedure for the Synthesis 6,8,9-Triamino-3-(4-substituted benzyl)-3H-imidazo[4,5-b]-1,8-naphthyridine-7-carbonitrile ***10***

DBU (0.53 mmol) was added to a solution of 2-amino-1,1,3-propenetricarbonitrile (0.61 mmol) in acetonitrile/ethanol mixture (3:1) and kept in an ice bath. The mixture was sitirred in an ice bath for 1 h. A suspension of cyanoformimdoyl imidazole **2a**,**b** (0.44 mmol) with the least amount of ethanol (2 mL) was added dropwise, and the mixture was stirred in an ice bath for 10 h (followed by TLC until the starting materials were consumed). the reaction mixture was allowed to stand at −10 °C for eight days, when the off-white solid was filtered and washed with ethanol and diethyl ether.

*6,8,9-Triamino-3-(4-methoxylbenzyl)-3H-imidazo[4,5-b]-1,8-naphthyridine-7-carbonitrile* (**10a**). (0.91 g, 2.52 mmol, 57%). m.p. 352–353 °C, IR (Nujol): 2211, 1678, 1594 cm^−1^. ^1^H-NMR (300 MHz, DMSO-*d*_6_): δ = 3.92 (s, 3H, OCH_3_), 5.35 (s, 2H, CH_2_), 6.69 (s, 2H), 6.81 (s, 2H), 7.32 (s, 2H), 7.47 (d, *J* = 8.6 Hz, 2H, Ar-H), 7.79 (d, *J* = 8.6 Hz, 2H, Ar-H), 8.96 (s, 1H). ^13^C-NMR (75 MHz, DMSO-*d*_6_): δ = 50.5, 55.6, 71.4, 110.6, 118.8, 121.4, 124.8, 128.7, 137.4, 139.6, 140.5, 141.8, 149.2, 151.7, 156.1, 159.9, Calcd for: C_18_H_16_N_8_O: C, 59.99; H, 4.48; N, 31.09. Found C, 59.86; H, 4.38; N, 31.23.

*6,8,9-Triamino-3-(4-methylbenzyl)-3H-imidazo[4,5-b]-1,8-naphthyridine-7-carbonitrile* (**10b**). (0.82 g, 2.38 mmol, 54%). m.p. 346–347 °C, IR (Nujol): 2226, 1686, 1594 cm^−1^. ^1^H-NMR (300 MHz, DMSO-*d*_6_): δ = 2.36 (s, 3H, CH_3_), 5.31 (s, 2H, CH_2_), 6.48 (s, 2H), 6.77 (s, 2H), 7.29 (s, 2H), 7.47 (d, *J* = 8.6 Hz, 2H, Ar-H), 7.79 (d, *J* = 8.6 Hz, 2H, Ar-H), 8.76 (s, 1H). ^13^C-NMR (75 MHz, DMSO-*d*_6_): δ = 21.2, 50.3, 71.4, 110.6, 118.8, 121.4, 124.8, 128.7, 137.4, 139.6, 140.5, 141.8, 149.2, 151.7, 156.1, 159.9. Calcd for: C_19_H_14_N_8_: C, 61.62; H, 3.81; N, 30.25. Found C, 61.57; H, 3.85; N, 30.31.

## 4. Conclusions

The reaction of 5-amino-4-(cyanoformimidoyl)imidazoles **4** with malononitrile or 2-amino-1,1,3-propene tricarbonitrile occurs selectively on the carbon of the cyanoformimidoyl substituent. Intramolecular cyclization of the adduct leads to 3*H*-imidazo[4,5-*b*]pyridines **5**, **8** and imidazo[1,8]naphthyridine derivatives **6** and **10**, depending on whether the reaction is carried out in the absence or in the presence of DBU, respectively. In absence of DBU, fused imidazole **5** and **6** synthesized from compound **4** and malononitrile, and 2-amino-1,1,3-propene tricarbonitrile upon the elimination of ammonia. In the presence of DBU, fused imidazoles **8** and **10** are generated from compound **4** and malononitrile or 2-amino-1,1,3-propenetricarbonitrile upon the elimination of hydrogen cyanide. Intramolecular cyclization of imidazole **7** occurs by heating in ethanol, in the presence of a base, such as triethyl amine, and successively in situ, in the case of intermediate imidazole **9**, as a result of the presence of highly substituted deficient alkene forcing the cyclization without a base, respectively.

## Abbreviations

DBU: 1,8-Diazabicyclo(5.4.0)undec-7-ene
